# A Novel Method of Autonomous Inspection for Transmission Line based on Cable Inspection Robot LiDAR Data

**DOI:** 10.3390/s18020596

**Published:** 2018-02-15

**Authors:** Xinyan Qin, Gongping Wu, Jin Lei, Fei Fan, Xuhui Ye, Quanjie Mei

**Affiliations:** 1 Department of Power and Mechanical Engineering, Wuhan University, Wuhan 430072, China; xyqin@whu.edu.cn (X.Q.); fei-fan@whu.edu.cn (F.F.); xhye@whu.edu.cn (X.Y.); meiquanjie@whu.edu.cn (Q.M.); 2 Key Laboratory of Hydraulic Machinery Transients, Ministry of Education, Wuhan University, Wuhan 430072, China

**Keywords:** autonomous inspection, transmission lines, cable inspection robot, LiDAR, position and orientation system, identification, positioning

## Abstract

With the growth of the national economy, there is increasing demand for electricity, which forces transmission line corridors to become structurally complicated and extend to complex environments (e.g., mountains, forests). It is a great challenge to inspect transmission line in these regions. To address these difficulties, a novel method of autonomous inspection for transmission line is proposed based on cable inspection robot (CIR) LiDAR data, which mainly includes two steps: preliminary inspection and autonomous inspection. In preliminary inspection, the position and orientation system (POS) data is used for original point cloud dividing, ground point filtering, and structured partition. A hierarchical classification strategy is established to identify the classes and positions of the abnormal points. In autonomous inspection, CIR can autonomously reach the specified points through inspection planning. These inspection targets are imaged with PTZ (pan, tilt, zoom) cameras by coordinate transformation. The feasibility and effectiveness of the proposed method are verified by test site experiments and actual line experiments, respectively. The proposed method greatly reduces manpower and improves inspection accuracy, providing a theoretical basis for intelligent inspection of transmission lines in the future.

## 1. Introduction

Power infrastructure is an important foundation for the national economy and people’s livelihood. Once power infrastructure goes wrong, it will cause large economic losses. Therefore, the state spends a great deal of manpower and material resources on inspection work every year [1]. As the economy grows, pylons and transmission lines are increasing rapidly. On the one hand, transmission lines have to expand to harsh environments, which are usually remote and complex, such as rugged mountains, resulting in difficult inspection work [2]. On the other hand, the power sector needs to use many compact towers with multi-loop to expand transmission capacity, which makes power line distribution and mutual inductance very complex, greatly increasing the difficulty of fault detection and ranging [3]. Inspection capabilities are facing these new challenges, pressing for an effectively autonomous inspection method using existing inspection equipment to reduce manpower and inspection errors and to improve inspection efficiency. Therefore, the study of autonomous inspection methods is of great practical significance [4,5].

Transmission line inspection aims to assess the operational condition of a transmission line and eliminate potential problems [6]. Due to long-term exposure in harsh conditions (e.g., large temperature difference, high humidity, and vegetation encroachment), many faults (e.g., damper slipping, broken strand, and electrical flashover) might occur, leading to large area blackout or potential forest fires [7]. Therefore, the tracking of the effects of fittings, wires, and the surrounding environment on transmission lines are important tasks for transmission line inspection. At present, inspection methods are mainly divided into four categories: manual inspection [8], vehicle inspection [9], airborne inspection [10], and robot inspection [11]. 

Manual inspection refers to observation of power lines and their surrounding conditions by the naked eye or a telescope. This method is labor- and time-intensive, costly, and inaccurate. Some power line segments subjected to topography factors cannot be inspected in areas it is difficult for inspection personnel/vehicles to access. Therefore, people strive to develop all kinds of advanced inspection methods to replace manual inspection. However, at present, manual inspection is still the most prevalent inspection method [12].

Vehicle inspection adopts a vehicle as a carrying platform with integrated GPS, INS, laser scanner, cameras, and other sensors to carry out inspection tasks along a power line [13]. Vehicle inspection is more flexible, as it can be loaded with some sizeable high-power inspection instruments to carry out precise inspection tasks [14]. However, this method is limited to power line inspection in a city [15]. 

Airborne inspection uses an aircraft as its carrying platform, which mainly includes three kinds, i.e., helicopter, fixed-wing UAV, and multi-rotor UAV. Among them, helicopter inspection is an efficient inspection method that is not restricted by region and can quickly complete inspection tasks without influencing power transmission [16]. However, helicopters are highly influenced by the complex operating environment. For example, many buildings are higher than the scanning height of a helicopter in urban areas, meaning that it is difficult to carry out high-quality inspection near a transmission line. Therefore, this inspection method is costly, weather-dependent, and hazardous. Currently, it is widely used in inspection tasks for ultra-high-voltage transmission lines [17]. Compared with helicopter inspection, fixed-wing UAV inspection has a lower cost. However, the disadvantages of fixed-wing UAV inspection are low accuracy, high technical threshold, and a time-consuming process for a large-scale inspection task [18,19]. Multi-rotor UAV has excellent flight stability and suspension capability, which is suitable for real-time observation and HD video recording of point-to-point on power line. However, the load of multi-rotor UAV is limited and it is difficult to carry a variety of inspection instruments at the same time. The short duration has limited its application to long-distance inspection [20].

Robot inspection uses a cable inspection robot as a motion platform. According to different inspection objects, it can be divided into substation robot inspection and CIR inspection. Substation robot inspection is used to replace manual inspection to execute substation inspection work [21]. CIR is a power robot traveling along ground wire, so it can reach rugged terrain, e.g., mountains, forests, and lakes, and it can carry out fine inspection in these regions [22,23]. CIR has some load capacity, which can be equipped with some inspection instruments. CIR can also resupply power online by the downhill-generated power or solar charge, which is suitable for automatic inspection in some complex environments [24].

In this study, autonomous inspection mainly includes three autonomous aspects: autonomous operation, autonomous target inspection, and autonomous positioning inspection. Autonomous operation is the ability to independently select motion path and automatically adjust movement according to path planning to reach various positions required by transmission line inspection, which is the foundation of the other autonomous applications [25,26]. Autonomous target inspection is the ability to automatically detect inspection targets or areas according to task planning [27,28]. Autonomous positioning inspection independently detects problems at specified points and provides positioning information.

Autonomous operation and autonomous target inspection are necessary and critical for UAV inspection and robot inspection. China Southern Power Grid has implemented the demonstration application of the automatic transmission line inspection using a fixed-wing UAV with multi-sensor in the complex environments [29]. However, it is not fully autonomous. A substation robot can autonomously operate in the substation and automatically judge the operation status and warnings of substation equipment [30]. CIR has broken through some key technologies, such as autonomous over-obstacle, autonomous positioning, autonomous fault diagnosis, and autonomous inspection [31]. After the necessary structural modifications of ground wires, the application of autonomous inspection has been completed on the whole transmission corridor. The automation levels of autonomous operation and autonomous target inspection have become increasingly mature.

However, autonomous positioning inspection of transmission line is still in its infancy. The abovementioned autonomous inspection methods use inspection instruments according to artificial planning and installation drawing to automatically take images or videos at fixed points. According to the pre-planning tasks, this will create a huge number of inspection images or videos. However, existing image processing methods for transmission line structure are not sufficient, due to the influence of complex background texture and light. They still rely on manual screening and positioning. Moreover, the missing inspection could have happened according to the pre-planning tasks, due to facilities shifts or random failures. These new challenges desperately need a platform with autonomous target inspection and autonomous positioning inspection to improve the intelligence level of transmission line inspection.

To sum up, through analysis of inspection methods and new demands of autonomous inspection, we propose a novel autonomous-positioning inspection method of transmission lines based on CIR LiDAR data. The main points of innovation are as follows:

1. Integrate advanced LiDAR technology and CIR technology to get unique CIR LiDAR data. CIR LiDAR, along a ground wire, would not be subject to topographic restriction like airborne LiDAR; CIR LiDAR can also scan objects at a closer range to obtain more fine-scale LiDAR data like vehicle-borne LiDAR. CIR LiDAR can also provide high-precision POS data that could represent the orientation and shape of power lines in a span segment.
2. Propose an autonomous inspection method based on CIR LiDAR data, combine the spatial information superiority of LiDAR and texture information advantage of image, and solve the practical problems in the autonomous inspection of transmission line at present.
3. Automatically identify inspection targets using point cloud and image processing technology in preliminary inspection, operate CIR along a ground wire to the specified positions based on 3D precise information of inspection targets, transfer angles or directions of the PZT camera to take high-quality images, and save the inspection information of targets into the inspection database. This will greatly improve the efficiency and accuracy of inspection, providing a theoretical foundation for intelligent inspection.

This paper is organized as follows. Section 2 describes the mechanical structure and hardware integration of the CIR autonomous inspection system. Section 3 proposes an autonomous inspection method using CIR LiDAR data. Section 4 illustrates two experiments to verify the effectiveness and feasibility of the proposed method. Section 5 provides the corresponding discussions. Conclusions are drawn in the last section.

## 2. Hardware

### 2.1. System Structure

**Figure 1:**
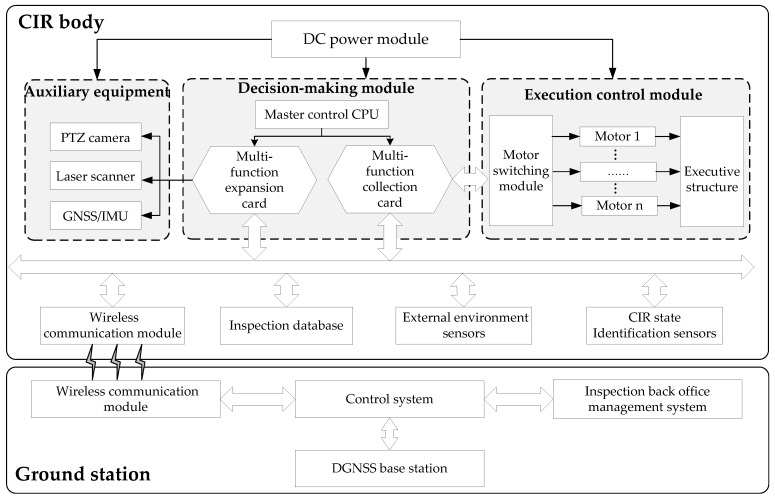
Overall design of CIR autonomous inspection system.

A structural diagram of the autonomous inspection system is shown in Figure 1, which is composed of a CIR body and a ground station. The CIR body part can be divided into a DC power module, a decision-making module, a control module, auxiliary equipment, a wireless communication module, an inspection database, external environment sensors, and CIR state identification sensors. The decision-making module is an industrially controlled computer system based on the PC104 bus to exchange data and communicate instructions through the bus and the multi-function card. The control module completes the control of motors. The auxiliary equipment includes a PTZ camera, a laser scanner, and DGNSS/IMU combination navigation. The inspection database stores all kinds of data, e.g., prior information on the transmission line, robot operation data, inspection target parameter information, inspection target images, etc. The sensors are divided into two categories: external environment detection and robot status identification, which ensures autonomous operation of CIR on the ground wire. The ground station is composed of a DGNSS base station, a control system, a back office management system, and a wireless communication module. The CIR body and ground station communicate wirelessly and are remotely monitored.

### 2.2. System Integration

System integration needs to fully consider the load capacity of CIR, power supply, installation space, etc. The configuration of the key components is shown in Table 1. In order to correct deviation errors caused by CIR at slow speed for a long time, we adopt the differential dual-antenna navigation mode. The size of the mobile station of the LiDAR system is about 255 mm long, 188 mm wide, and 150 mm high. The total weight (excluding cables) is 3.46 kg, and the maximum power is 22.5 W. The PTZ camera is SNC-WR630 (SONY) with a weight of 1.7 kg.

**Figure 2:**
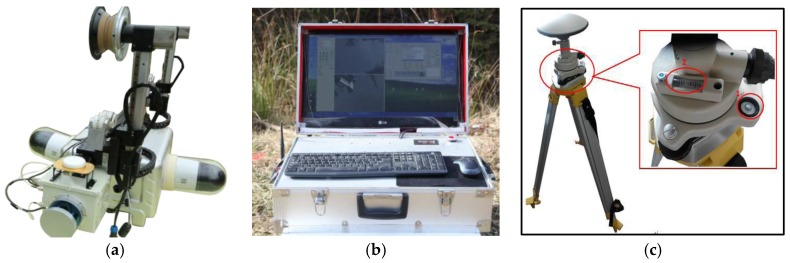
Photos of the autonomous inspection system: (**a**) CIR body; (**b**) base station; (**c**) GNSS base station.

Since CIR moves along the ground wire to perform inspection tasks, power lines and trees are usually lower than CIR’s position. The LiDAR host is installed on the end of CIR below the crossbeam. The two mobile antennas are fixed at both ends of CIR’s crossbeam and extend outward to meet the baseline length of the dual-antenna pattern. The PTZ cameras are installed on both sides of the robot to avoid interference. Figure 2 gives photos of the autonomous inspection system.

## 3. Methodology 

**Figure 3:**
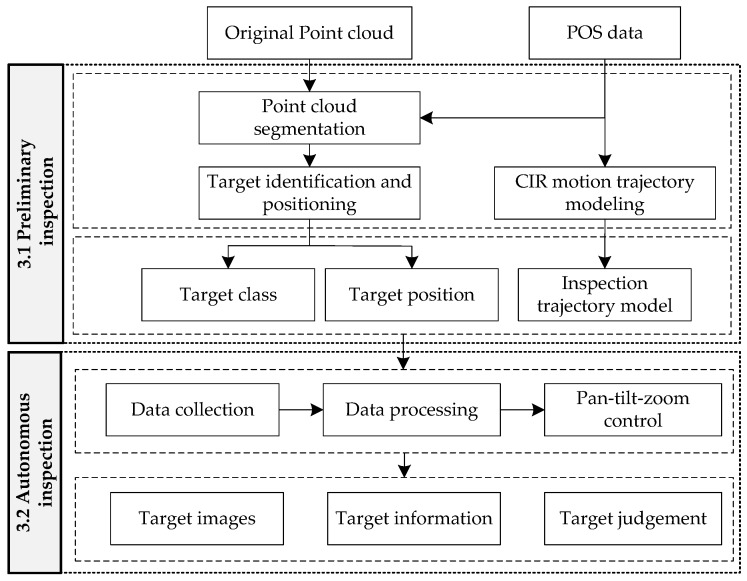
Technical framework of the proposed autonomous inspection method.

We propose a novel method to autonomously inspect transmission lines based on CIR LiDAR data, as illustrated in Figure 3, which mainly includes two steps: preliminary inspection and autonomous inspection. 

### 3.1. Preliminary Inspection

#### 3.1.1. Point Cloud Segmentation

CIR LiDAR resolves the combined navigation data through an extended Kalman filter to obtain the accurate motion trajectory of CIR, i.e., POS data. The POS data and laser data are converted to a reference frame to get the coordinates of scene point clouds.

**Figure 4:**
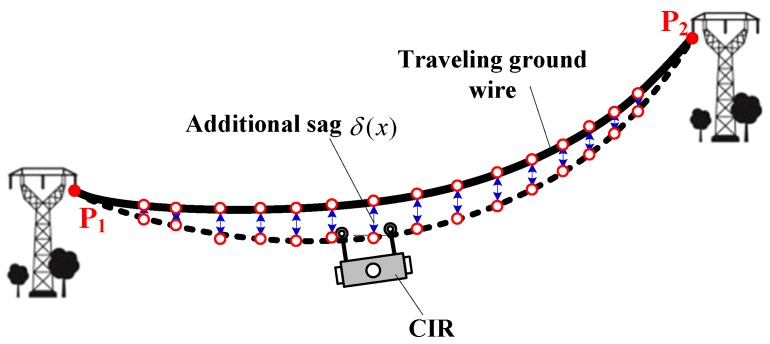
Additional sag of the walking ground wire.

CIR fully moves along the ground wire, which produces a precise trajectory and obtains point clouds at the same time. The trajectory points and scene point clouds derived from different collection sources are naturally separated, which can reflect the overall orientation of all power lines in the same span segment (refers to the segment of wire between poles/towers) and the segmentation information. Therefore, a point cloud segmentation method based on a previous study [32] is established to extract the point cloud of each transmission line and its surrounding points. The main steps are as follows:

(1) Point cloud partition of a span segment based on POS data. In the process of inspection, the motion of CIR presents uphill–downhill–uphill. According to work characteristics and body angle data measured by obliquity sensors, suspension points of ground wire are assigned. In this way, multi-segment transmission lines can be partitioned into single segment transmission lines in point cloud data.
(2) Ground point filtering by the elevation threshold value of POS. In addition to transmission line points of a span segment, there are also a few ground points and remaining surface points. The transmission line points in a span segment are generally higher than the ground points, so the POS elevation threshold is defined to quickly remove all ground points and most of the remaining surface points.
(3) POS-based structured partition. The filtered transmission line point cloud and POS data are projected to the optimal coordinate plane (OCP) by the coordinate transformation. Then, we establish the extraction model by fitting POS data. The ground wire is subject to additional sag due to the additional weight of CIR, as illustrated in Figure 4. The fitting POS-based model is revised with the additional sag function, δ(x) [32]. Through the POS extraction model, transmission line point clouds are divided into single lines in a span segment, at the same time retaining waiting inspection target point clouds around the line.

#### 3.1.2. Target Identification and Positioning 

**Figure 5:**
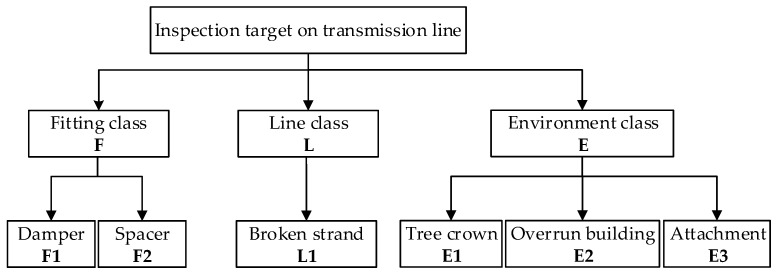
The classification of inspection targets on a transmission line.

The purpose of the inspection is to check fittings (e.g., dampers, spacers) on a transmission line to find abnormal points and assess the surrounding environment of lines, which enables power personnel to quickly arrive at the fault points for maintenance. The inspection target on a transmission line is generally classified into three kinds, as shown in Figure 5.

Through analysis, inspection targets are attached to a transmission line or its surroundings. The different types of inspection targets have their own characteristics, as illustrated in Table 2. 

**Figure 6:**
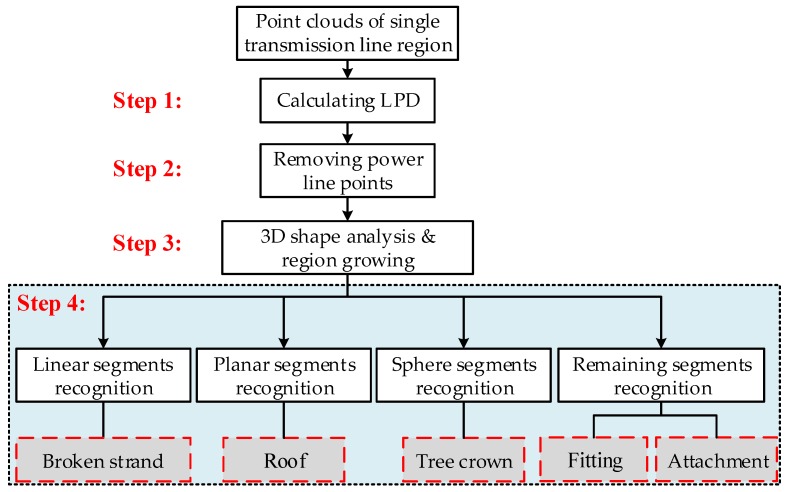
The algorithm flow chart.

For a point cloud of a single transmission line, a hierarchical classification strategy is used to identify different inspection targets using the distribution characteristics of a point cloud and the geometric characteristics of targets. The algorithm flow is shown in Figure 6.

The specific procedures are listed as follows.

Step 1: Calculating local point density (LPD) of each point in a single transmission line region by Equation (1):

(1) $$D_{i}=\frac{k}{\pi d^{2}{}_{k}}\begin{array}{cc} & \left(i=1,\cdots ,N\right) \end{array},$$

where *k* is the number of neighboring points to the point in question (in this paper, *k* = 16); $d_{k}$ is the distance between the point in question and the farthest point in *k*-nearest neighbor.

Step 2: Removing power line points by the threshold of LPD. The threshold of removing power line points is determined from the density histogram of the point cloud in a single transmission line region. The dominant peak corresponding to power line points is automatically identified, from which LPD is determined. After removing power line points, the residual point clouds include the inspection objects in the partition.

**Figure 7:**
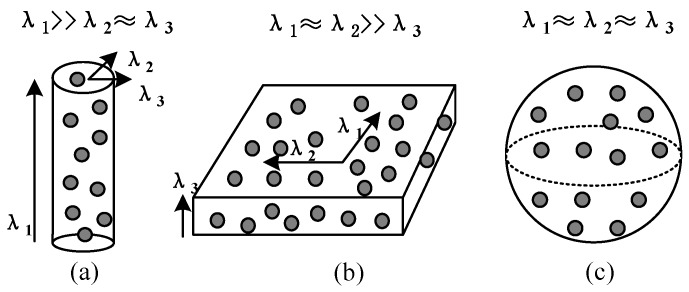
Illustration of the saliency features: (**a**) Linearity; (**b**) planarity; (**c**) sphericity.

Step 3: Use local neighborhood statistics derived from principle component analysis (PCA) to analyze the spatial distributions of the residual point clouds. The optimal neighborhood radius of each point is determined by the neighborhood adaptive method [33]. The data in the neighborhood are adopted PCA to obtain the eigenvalues of the local point cloud, $\lambda_{1}$,$\lambda_{2}$,$\lambda_{3}$. The distributions of different target eigenvalues have obvious differences, determining the spatial distribution of the local point cloud. The relationships among these three eigenvalues indicate the distributions of the 3D points: if $\lambda_{1}\gg \lambda_{2}\approx \lambda_{3}$, the points are linear distribution; if $\lambda_{1}\approx \lambda_{2}\gg \lambda_{3}$, the points are planar distribution; if $\lambda_{1}\approx \lambda_{2}\approx \lambda_{3}$, the points are discrete distribution as illustrated in Figure 7 [34,35]. 

Then, region growing is employed to segment the points identified as belonging to the shaped neighborhood. The region growing algorithm randomly chooses a seed point from the identified points within 0.2 m 3D Euclidean distance. Then, we divide the LiDAR returns into four disjoint subsets, i.e., “linear” points, “planar” points, “sphere” points, and remaining “block” points.

Step 4: Recognizing inspection targets from the above four subsets. About linear segments, there are two types of objects. One is a power line, not completely eliminated in Step 2, which appears as straight linearity. The other is a broken strand, which appears as curvilinear linearity. Therefore, curvilinear points are identified as broken strands, at the same time removing straight linear points according to the curvature. About planar segments, these points having vertical angles bigger than the threshold (85°) are identified as a roof; otherwise they are labeled as remaining points. Sphere segments having an XY projected area bigger than the threshold are identified as tree crown; otherwise they are labeled as the remaining points too.

**Figure 8:**
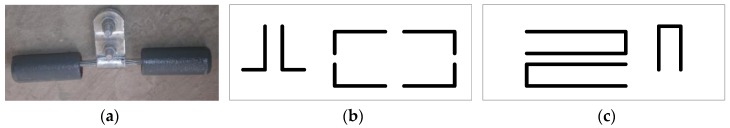
Feature samples of damper: (**a**) Photo of damper; (**b**) 2AS local contour; (**c**) 3AS local contour.

Finally, there are two types of objects, fitting and attachment, formed by the remaining points. Since the fittings are artificial facilities, they generally have definite contour features. So a local contour feature (LCF) algorithm is used to identify the fittings [36,37]. Firstly, the points are projected to the XZ plane, and the edge image and contour chain code of point clouds are used to preprocess images. Secondly, the contour chain code is used to extract independent straight line segments, and the adjacent independent linear segments are grouped together to form local contour characteristics, kAS. Thirdly, the detected local contour features are clustered and coded. Fourthly, the local contour features are traversed by the established multi-combination conditions. The samples of local contours of the damper are shown in Figure 8 [38,39]. Other points are recognized as attachments.

After identification of all kinds of targets, map the obtained datasets of each object back to 3D space, get the point cloud blocks of the corresponding inspection targets, and calculate the mass centers of the point clouds. Finally, the identification results and coordinates of the mass centers are stored in the inspection database as the waiting-inspection target information.

#### 3.1.3. CIR Motion Trajectory Modeling 

The POS can accurately reflect the trajectory of a ground wire. In order to determine a CIR’s position relative to the tower, it is necessary to build up a motion trajectory equation of CIR.

**Figure 9:**
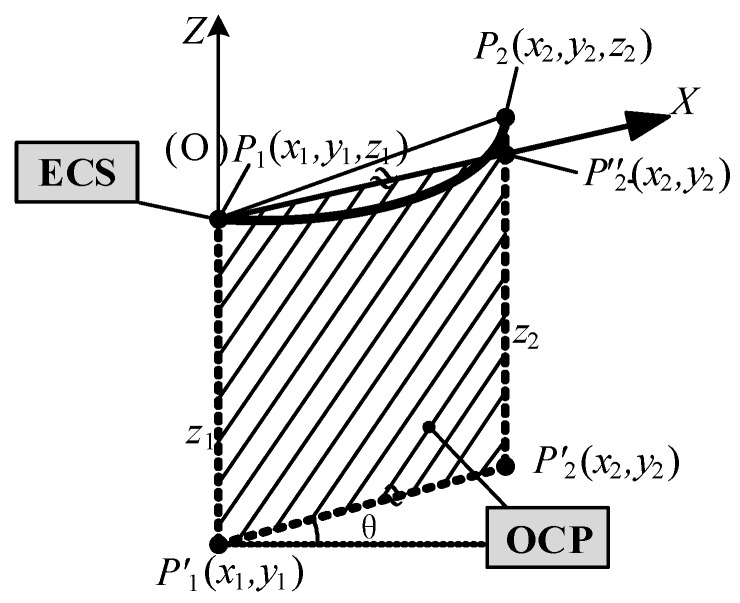
The construction of OCP and ECS.

The suspension points (i.e., P_1_ and P_2_) of the ground wire on the tower are determined by point cloud segmentation using the POS data, as illustrated in Figure 9. In a span segment, the suspension point of litter-number tower P_1_ is considered as the origin O of the engineering coordinate system (ECS); the elevation of POS data is applied as the Z-axis of ECS. The two suspension points (P_1_ and P_2_) are projected to the horizontal plane, generating two points (P_1′_ and P_2′_). The optimal coordinate plane (OCP) can be determined by lines P_1′_P_2′_ and P_1_P_1′_. In OCP, the line P_1_P_2′_’ is parallel to the line P_1′_P_2′_ through point P_1_, intersecting line P_2_P_2′_ at point P_2′_’. The line P_1_P_2′_’ is defined as the *x*-axis of ECS.

POS data fit curves with the optimal catenary model. In order to simplify the operation, the nonlinear catenary equation is simplified to a linear polynomial according to the Lagrange polynomial principle. The curve model fitting POS data is obtained by Equation (2):

(2) $$Z=Ax^{2}+Bx+C.$$

The following results can be obtained after preliminary inspection:

- How many waiting-inspection targets (refers to these inspection targets that need to be inspected again in the autonomous inspection) on transmission lines are there?
- Each waiting-inspection target belongs to one transmission line;
- The identification result and space position of each inspection target;
- The trajectory model of the ground wire.

Next, the autonomous inspection of CIR will be completed using the abovementioned information data.

### 3.2. Autonomous Inspection

#### 3.2.1. Coordinate System Modeling

(1) Inspection Coordinate System

**Figure 10:**
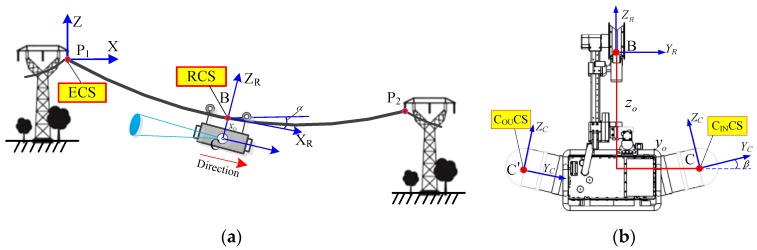
Coordinate systems of the autonomous inspection: (**a**) ECS and RCS; (**b**) C_IN_CS and C_OU_CS.

Inspection coordinate system is built in order to parameterize CIR’s position on a transmission line relative to the reference point and its own state, which mainly includes four coordinate systems, i.e., the reference point, CIR body, and the two PTZ cameras, as shown in Figure 10. Through the mathematical relations between each coordinate system, CIR can be executed the pose transformation and control the position of CIR in real time.

ECS is the reference point coordinate system; RCS is the CIR body coordinate system, and the origin of RCS is defined as the mid-point of the two pressure wheels. C_IN_CS and C_OU_CS are two PTZ coordinate systems, i.e., inward view and exterior view, respectively, which have the specified geometric centers of the cameras as their origins. *α* is the angle between the robot on the ground wire and the horizontal plane. *β* is the installation angle between the PTZ camera and the horizontal plane. ECS is a fixed-coordinate system; RCS and CCS are mobile-coordinate systems.

(2) Coordinate Transformation

In order to direct a PTZ camera towards waiting-inspection targets to take high-quality images, coordinate transformation needs to be completed between the positions of inspection targets and the reference according to CIR’s inspection position.

CIR obtains the inspection target relative to ECS position information (*X*’, *Y*’, *Z*’) and posture parameters data in the inspection database. The position information of inspection target is converted to CCS, getting the new 3D coordinate (*X*, *Y*, *Z*) as defined in Equation (3):

(3) $$\left[\begin{array}{c} X\\ Y\\ Z\\ 1 \end{array}\right]=\left[\begin{array}{cccc} 1 & 0 & 0 & 0\\ 0 & \cos\beta & -\sin\beta & 0\\ 0 & \sin\beta & \cos\beta & 0\\ 0 & 0 & 0 & 1 \end{array}\right]*\left[\begin{array}{cccc} 1 & 0 & 0 & \Delta x\\ 0 & 1 & 0 & 0\\ 0 & 0 & 1 & \Delta z\\ 0 & 0 & 0 & 1 \end{array}\right]*\left[\begin{array}{cccc} \cos\alpha & 0 & \sin\alpha & x_{0}\\ 0 & 1 & 0 & y_{0}\\ -\sin\alpha & 0 & \cos\alpha & z_{0}\\ 0 & 0 & 0 & 1 \end{array}\right]*\left[\begin{array}{c} X^{\prime }\\ Y^{\prime }\\ Z^{\prime }\\ 1 \end{array}\right],$$

where *β* = 13°; Δ*x* and Δ*z* are the horizontal distance and vertical distance relative to the reference point, respectively. *x*_0_, *y*_0_, and *z*_0_ are the offsets of the CIR body. The sign symbol of *y*_0_ represents the different cameras on the inside and outside, i.e., inward view with a positive sign, and exterior view with a negative sign. 

#### 3.2.2. Autonomous Inspection Planning

Before the autonomous inspection, the guidance information required by CIR is established through inspection planning, which mainly includes database structure model and inspection sequence flows of the waiting-inspection targets.

**Figure 11:**
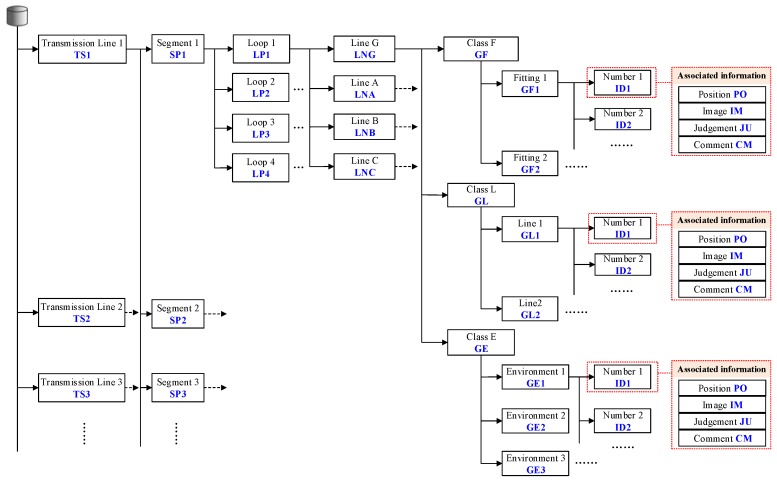
The schematic diagram of the database structure of a waiting-inspection target.

The database structure of the waiting-inspection targets is based on the prior information of transmission line and the results of preliminary inspection, as shown in Figure 11. CIR inspects different lines: Transmission Line 1, Transmission Line 2, etc. For any one of the lines, the transmission line is divided into Segment 1, Segment 2, etc. Segments is divided into Loops 1–4. A loop is composed of a ground wire, A-phase, B-phase, and C-phase. The waiting-inspection targets include fitting class, line class, and environment class. Each waiting-inspection target is assigned a number by certain rules. The numbered waiting-inspection targets have associated information, such as position (PO), image (IM), judgment (JU), comment (CM), etc. 

**Figure 12:**
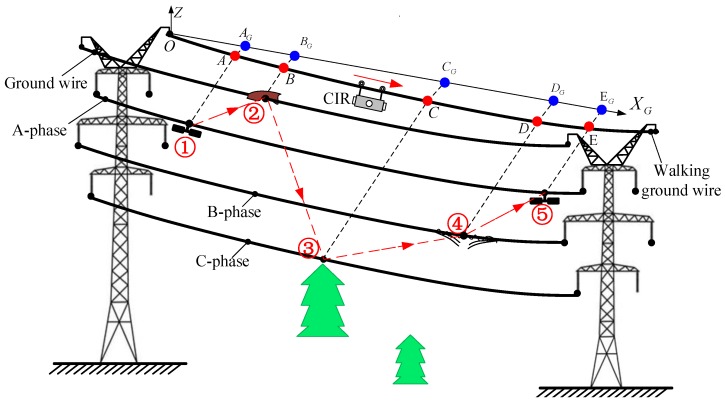
The schematic diagram of the generated inspection sequence.

The inspection sequence can be generated according to the database structure model of inspection targets. Figure 12 illustrates how to generate an inspection sequence. Firstly, use the segment number to search the inspection database to obtain motion parameters and inspection information on the current segment. According to the X-coordinate in ECS, the waiting-inspection targets are assigned a number in order.

**Figure 13:**
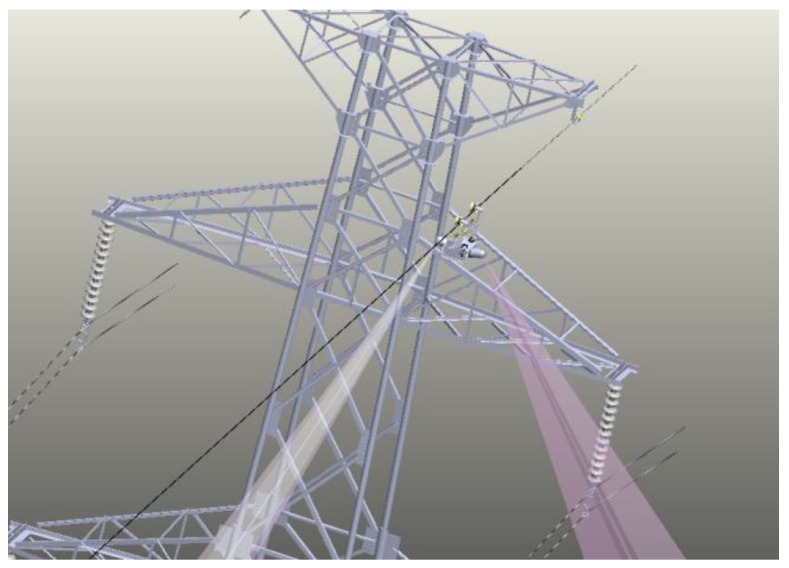
The schematic diagram of the autonomous positioning inspection.

When CIR autonomously inspects the *k*-th span segment, the inspection sequences are obtained in turn, as shown in Table 3. When approaching an inspection target, the CIR will slow down, stop at the inspection point, and adjust the PTZ camera to take images. For the inspection targets on the same side of the walking ground wire, the exterior-view camera is deployed to take images; for the opposite of the walking ground wire, the inward-view camera is used to take images, as illustrated in Figure 13. Finally, the captured images are stored in the inspection database corresponding to the inspection target parameter IM, and the final judgment result and comment are also stored in the inspection target parameters JU and CM, respectively, by manual comparison.

#### 3.2.3. Autonomous Inspection Procedure

**Figure 14:**
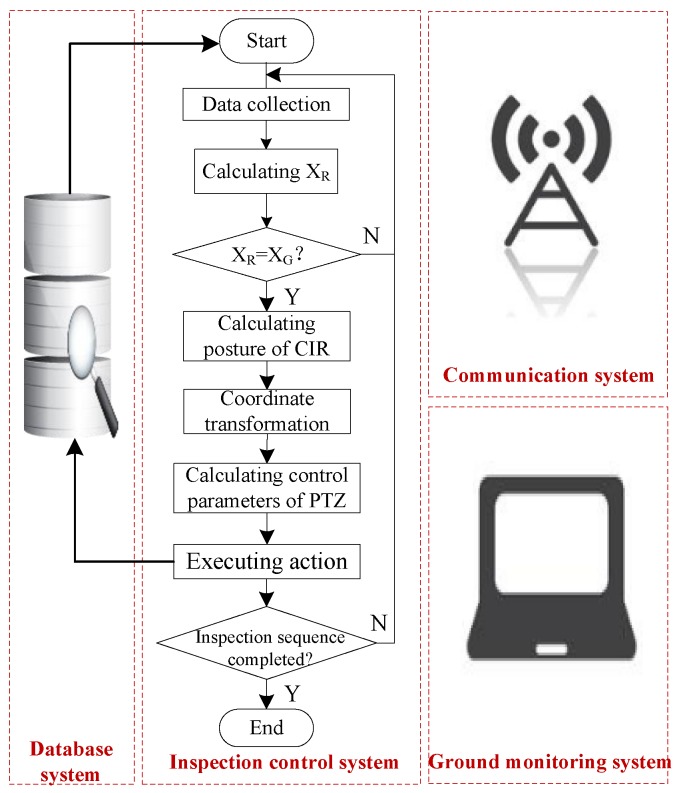
The block diagram of the inspection control.

The inspection procedure is shown in Figure 14, involving an inspection database system, an inspection control system, a communication system, and a ground monitoring system. The inspection control system is the core, which mainly executes data collection, data processing, PTZ camera control, etc.

The specific inspection procedures are listed as follows.

Step 1: Obtain the inspection planning in the inspection database. CIR starts to autonomously operate. In the process, CIR constantly collects multi-sensor data.

The coordinates *X_R_* of CIR relative to ECS are obtained through multi-sensor data fusion. The multi-sensor data include measuring the angle α, mileage of walking wheel $RO_{i}$ and range of pressure wheel ${PO}_{j}$, as shown in Equation (4):

(4) $$\begin{array}{l} {RO}_{i}=\frac{2\pi R\Delta C_{i}}{Km}\\ PO_{j}=\frac{2\pi r\Delta C_{j}}{n} \end{array},$$

where *R* is the radius of the walking wheel; ${\Delta C}_{i}$ is the value of the walking wheel encoder; *K* is the baud rate of the motor; *m* is the reduction gear ratio; *r* is the radius of the pressuring wheel; ${\Delta C}_{j}$ is the value of the hall sensor of the pressuring wheel; and *n* is the number of magnetic steel parts in the pressuring wheel. After wheel-slippage optimization compensation, CIR walking mileage can be expressed as in Equation (5):

(5) $${WO}_{e}=(1-K_{s})(1-sign(\alpha \cdot \omega )\cdot K_{e}\left|\alpha \right|)RO_{i}+K_{s}K_{p}PO_{j},$$

where *ω* is the angular velocity of the walking wheel; $K_{s}$ is the state scale factor; $K_{e}$ is the empirical coefficient; and $K_{p}$ is the compensation coefficient of the pressuring wheel.

Step 2: The mathematical model of transmission line is catenary. If the collection spacing is small enough, CIR walking mileage $WO_{e}$ can be considered an arc. The coordinate $x_{R}$ of CIR relative to ECS can be defined as in Equation (6):

(6) $$x_{R}=\sum_{i=1}^{n}{WO}_{e}{}_{i}\cos\left(\alpha_{i}+\alpha_{i+1}\right)/2,$$

where $WO_{e}{}_{i}$ is the CIR walking mileage in *i*^th^ collection spacing; and $\alpha_{i}$ and $\alpha_{i+1}$ are the inclinations of two adjacent collections.

Step 3: Determine if CIR is at the right inspection position by comparing $x_{R}$ and $x_{G}$. If it arrives at the positon, CIR will stop and lock two arms on the transmission line. The sensors get the posture of CIR, and the $z_{R}$ of CIR is obtained using the CIR motion trajectory model; if not, CIR will keep on moving.

Step 4: Substitute the posture parameters of CIR and the position parameters of the inspection targets into Equation (8) to obtain the coordinates of the inspection targets in CCS.

**Figure 15:**
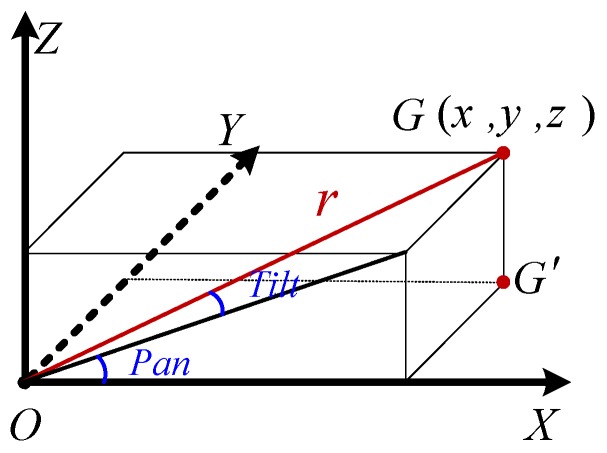
The diagram of 2DOF rotation angle of PTZ camera.

Step 5: Calculate the motion adjustment parameters of PTZ camera. The PTZ camera has two rotation degrees of freedom, as shown in Figure 15. The *G* point is the inspection target. Equation (7) is the adjustment parameter model of the PTZ camera.

(7) $$\begin{array}{l} Pan=\arctan(z/x)+s\_Pan\\ Tilt=s\_tilt-\arctan(y/\sqrt{x^{2}+z^{2}})\\ r=\sqrt{x^{2}+y^{2}+z^{2}} \end{array},$$

Step 6: Rotate to the specified angle of PTZ camera, choose the right focal length and magnification according to the *r* value, take the images, and store the image files in the inspection database.

Step 7: Judge whether all inspection sequences are executed. The inspection task will stop once all inspection sequences have been completed.

## 4. Results

### 4.1. Test Site Experiment

**Figure 16:**
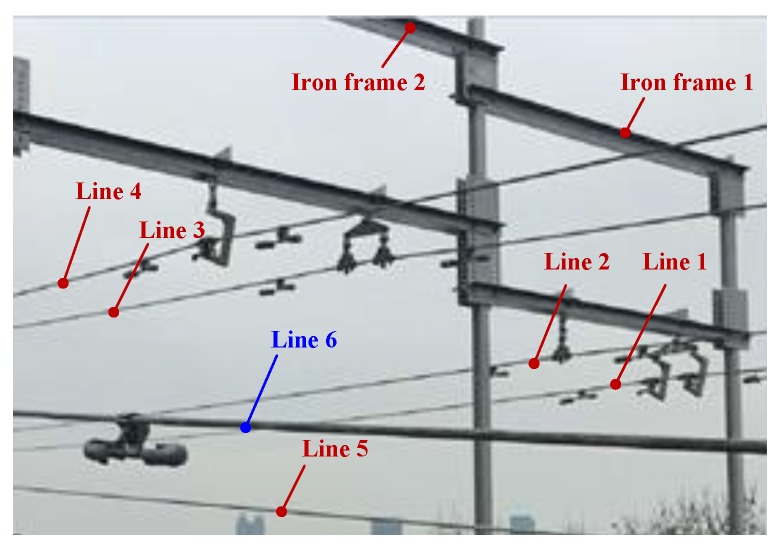
Photo of the test site.

In order to verify the effectiveness of the proposed method, we have constructed a test site to carry out experiments. The test site is 50 m in length and 30 m in width, located on the top floor of a building, which is constructed six parallel LGJ-95 lines: Lines 1–4 on the first layer and Lines 5–6 on the second layer. CIR is hung on Line 6 to perform the inspection task. Figure 16 is a photo of the test site.

**Figure 17:**
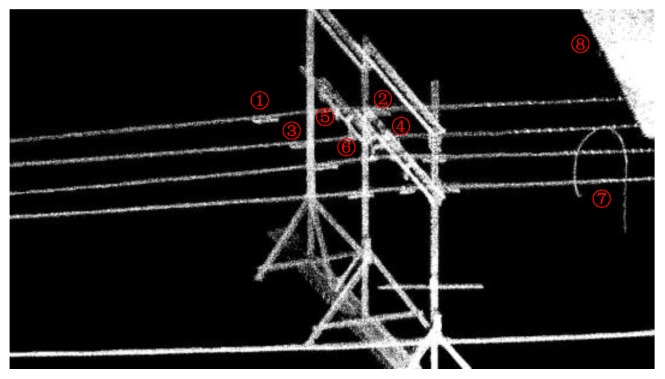
The original point cloud of the test site.

Figure 17 shows the point cloud of the test site collected by CIR LiDAR system. The average point density is 6097 per square meter, which is very dense over three orders of magnitude relative to ALS. These point clouds of lines and their surroundings are fine, and the density and geometric characteristics of the dampers are obvious, as shown in Figure 17.

The distribution of these power lines does not strictly comply with the construction specifications due to the size limit of the test site. The starting point of CIR is to establish ECS. In this paper, Iron frame 1 and Lines 1–2 are used as examples to illustrate the autonomous inspection process. In Figure 17, Targets 1 and 2 are two dampers on Line 1; Targets 3 and 4 are also two dampers on Line 2; Targets 5 and 6 are two hanging panels on the Iron frame 1, which is to simulate attachment on the line; Target 7 is used to simulate a random attachment on the line; Target 8 is the roof of the building to simulate the overrun building. 

**Figure 18:**
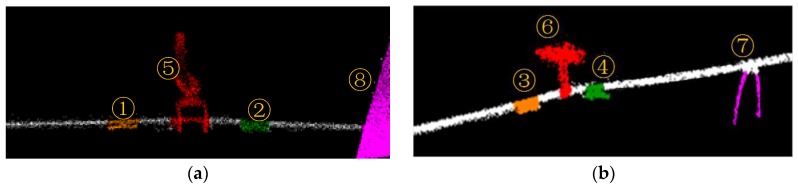
Classification results: (**a**) classification point clouds of Line 1; (**b**) classification point clouds of Line 2.

The point cloud is separated into each line based on the POS data. The spatial distribution and geometrical structure of the point cloud are used to identify and locate the inspection targets on Lines 1 and 2. Figure 18 illustrates the classification point clouds of these inspection targets on Lines 1 and 2.

All waiting-inspection targets are numbered along the line relative to the reference point, generating the inspection sequence. The PTZ camera is autonomously adjusted to take images that are stored into the inspection database for inspection judgement. Table 4 gives inspection information on Lines 1 and 2.

### 4.2. Actual Line Experiment

**Figure 19:**
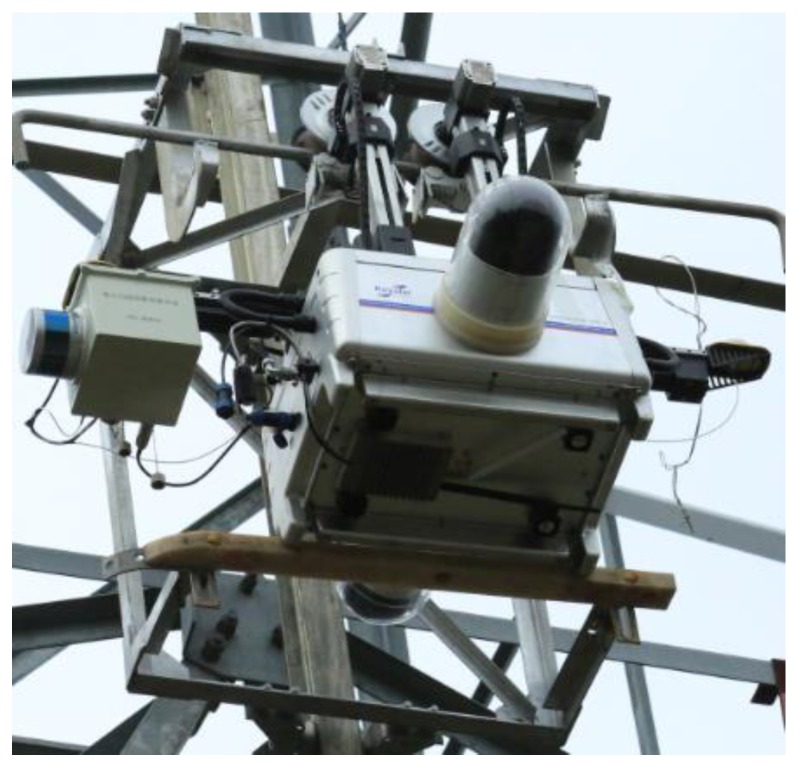
The automatic up-line device.

**Figure 20:**
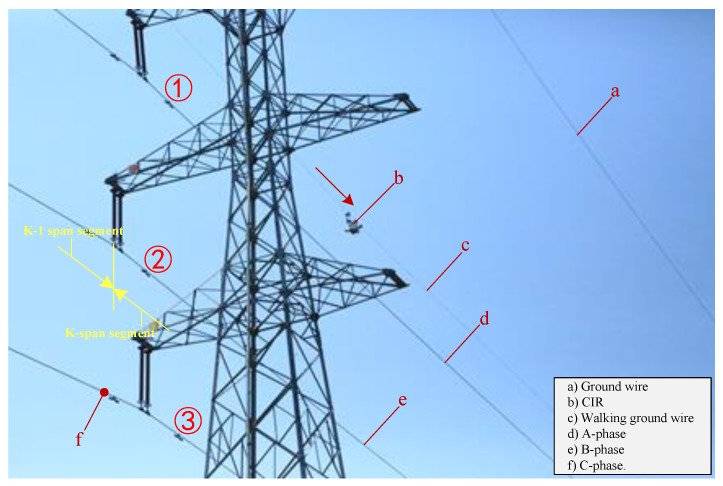
A photo of the actual line.

To verify the feasibility of the proposed method, we carried out many experiments on the actual line, which is located in the Changbai Mountains, Jilin Province. The actual lines are loaded with 220 KV (high voltage). The autonomous inspection system integrating a CIR LiDAR system and PTZ cameras is transported on the walking ground wire through the automatic up-line device, as shown in Figure 19. There are five transmission lines on the tower in Figure 20: two ground wires at the top layer and the three-phase transmission lines under the same side of the walking ground wire. Targets 1–3 is the three dampers on the transmission lines in k span segment. There are no other abnormal conditions (e.g., attachment, broken strand) in the segment through observation.

**Figure 21:**
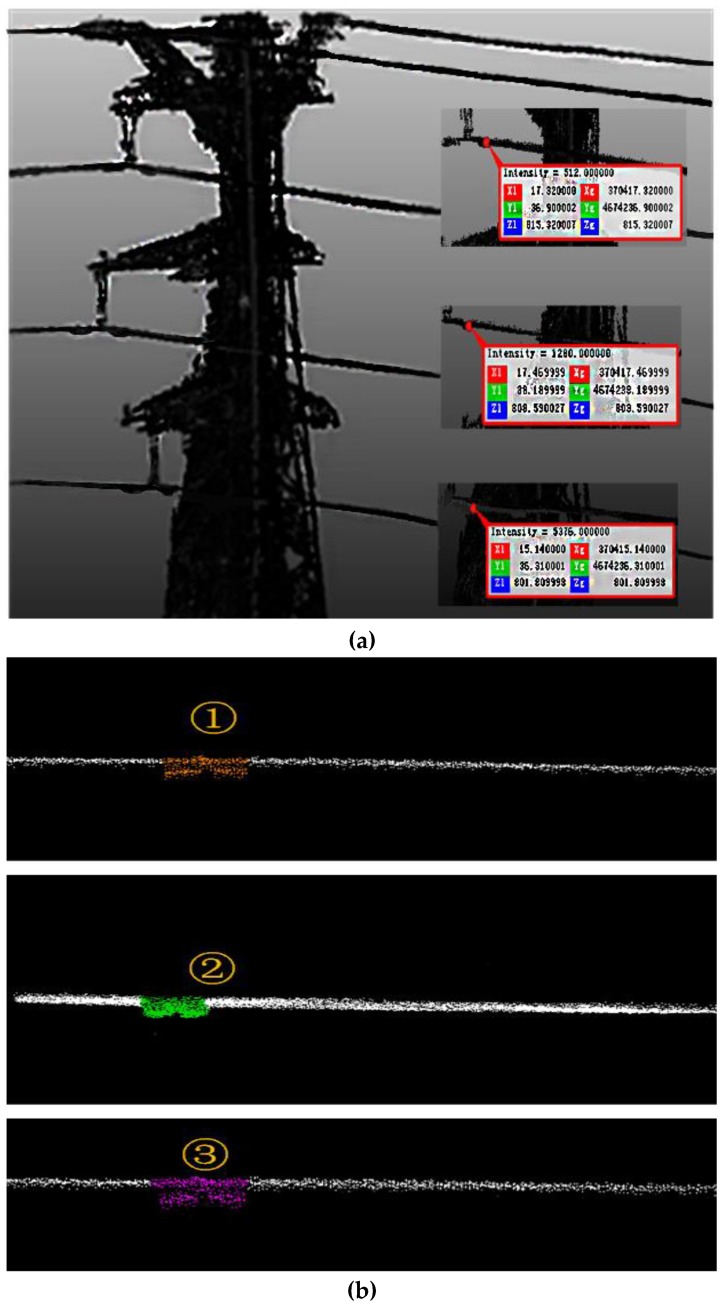
Point cloud results: (**a**) The point cloud; (**b**) the classification results.

Figure 21a shows the point cloud of the actual line, which can be seen as dampers and other fittings on the transmission line. The suspension point on the walking ground wire is set as a reference point. The three phase conductors are extracted by the structured partition. The maximum local density of a single line is extracted from the point cloud, as shown in Figure 21b, in which one can clearly see the contour feature of the damper. Therefore, the waiting-inspection targets are identified as damper (GM) by the image processing method, and the accurate positions of targets are determined by calculating the mass center of the damper point cloud on the line.

All waiting-inspection targets are numbered along the motion direction relative to the reference point to generate the inspection sequences. The PTZ camera is autonomously adjusted to take images that are stored into the inspection database for inspection judgement. Table 5 gives inspection information of targets in the inspection database, showing good accuracy for autonomous inspection. 

## 5. Discussion

The idea of the proposed method is derived from the rapid development and application of CIR technology and LiDAR technology. The most striking characteristic of CIR is inspection transmission line along a ground wire. Its inspection perspective is different from the aerial view of an aircraft, which is wide but not very detailed. It is also different from vehicle inspection or artificial inspection from ground to sky that could be frequently covered by trees, buildings, etc. LiDAR technology has been rapidly developing in recent years, especially in terms of the light weight and cost-effectiveness of hardware, which makes it possible to carry LiDAR on CIR to propose a new method of transmission line inspection. The CIR LiDAR data, combining the characteristics of CIR and LiDAR, are very distinctive data: Firstly, CIR LiDAR slowly scans the transmission line at close range to obtain a more detailed scene point cloud; secondly, POS data can obtain the model of ground wire, which can also reflect the orientation of the other transmission lines in the same span segment. Therefore, it can be used for post-processing of a transmission line point cloud. Thirdly, CIR could inspect the same working conditions along the fixed path. The inspection path and the scene point cloud have good repeatability, providing a new perspective for autonomous positioning inspection. These characteristics of CIR LiDAR data provide a strong technological support for detailed inspection of transmission lines.

The main contribution of the proposed method is to enhance autonomous inspection of transmission lines, which includes autonomous operation, autonomous target inspection, and autonomous positioning inspection. At present, the mentioned autonomous inspection refers to taking images or videos at the given points according to the pre-planning tasks. Autonomous positioning inspection is when the carrying platform can find the problems on a transmission line, provide positioning information of targets, operate the platform to reach the specified positions, and supplement inspections using the other testing instruments. In this paper, a single transmission line is extracted by the POS extraction model. Using space distribution characteristics and the geometrical structure characteristic of waiting-inspection targets, a hierarchical inspection strategy is proposed to identify inspection targets. The proposed method takes full advantage of the LiDAR 3D information and visible light texture information, improving the accuracy and efficiency of inspection, especially in reducing human labor in the post-processing phase.

The proposed method provides improvement to practical applications in three aspects. Firstly, the proposed method needs to conduct two inspections online because CIR LiDAR data should be generated and processed offline. In order to maintain the consistency of the inspection conditions, the interval between the two inspections should not be too long. In the future, we hope that CIR could complete the whole process online with the development of LiDAR software technology. Secondly, the load capacity of CIR is limited to select the large-scale instruments that can obtain higher-quality CIR LiDAR data. We want to use lighter instruments to obtain better point clouds with the development of LiDAR hardware technology. Thirdly, because the position of the tower is fixed, the autonomous inspection only needs to determine the relative positions of the inspection targets in a span segment. Therefore, it is possible that the visual odometer could replace the DGNSS to provide navigation data for the laser scanner.

## 6. Conclusions

In the study, we propose a novel method of autonomous inspection for transmission lines using CIR LiDAR data. The main conclusions of our study are as follows:

(1) CIR can become a new type of carrying platform to collect LiDAR data. Because CIR always moves along the ground wire, the POS data can be used to deal with a transmission line point cloud and build up a CIR motion trajectory model. In addition, CIR can closely scan transmission line corridors, so the point cloud is very dense. The abnormal points can be clearly presented in the transmission line point cloud.
(2) The proposed method mainly includes two inspection steps. The preliminary inspection can find the abnormal points through point cloud distribution, and use a hierarchical classification strategy to determine their classes and 3D positions. The autonomous inspection can generate the inspection sequences according to the inspection target information obtained in preliminary inspection, operate CIR to reach the specified points in accordance with inspection planning, and take images of the inspection targets with the PTZ cameras. Therefore, in the post-processing phase, it is no longer necessary to extract useful information from the many inspection images or videos, but only to make a final judgement by contrasting images at specified points and preliminary inspection results. This method can effectively improve the intelligence level of the current transmission line inspection, greatly reducing manpower and improving inspection accuracy.
(3) In test site experiments, we designed eight inspection targets including three classes of transmission line inspection. Experimental results show that CIR can move to the specified set points in turn and take clear images to assist the artificial judgement for the wrong classifications or hard identifications, which verifies the effectiveness of the proposed method. Actual line experiments prove that the autonomous inspection system can collect the required data. CIR can inspect the three dampers in the span segment and transfer the PTZ cameras to take images, which verifies the feasibility of the proposed method.

Further research work should mainly focus on two aspects. One is that it is more difficult to set the effective thresholds of LPD if the features of abnormal points are not obvious. The point cloud classification algorithm should be further improved to obtain better identification ability. The other is that PTZ cameras sometimes cannot achieve ideal image quality for the inspection targets in actual line experiments. We will carry out a multi-factor analysis on the control of the PTZ cameras.

## Figures and Tables

**Table 1:** Key components of CIR autonomous inspection system.

Component Name			Mode	Amount
CIR	CIR body		Walking through mode	1
	PTZ camera		SNC-WR630	2
	LiDAR	Laser scanner	VLP-16	1
		POS system	APX-15UAV	1
		Mobile-end antenna	HX-CA7606A	2
		Direction-finding receiver	FLEX6-D2S-Z00-00N	1
		Memory card	128 G	1
CIR base station	CIR base station		Customization	1
GNSS base station	Reference station receiver		SDI-228	1
	Reference station antenna		HY-BGLRC08R	1

**Table 2:** Characteristic rules of inspection targets.

Class Name	Inspection Target		Feature Description	Density	Contour Feature
Fitting (F)	Damper	F1	Artificial facility Attached to the power line	Dense	“T” shape
	Spacer	F2	Artificial facility Attached to the power line	Dense	“X” shape
Line (L)	Broken strand	L1	Attached to the power line	Dense	Curve shape
Environment (E)	Tree crown	E1	Under the power line Large size	Dense	Sphere shape
	Overrun building	E2	Under the power line Large size	Dense	Planar shape
	Attachment	E3	Attached to the power line Small size	Dense	Irregular shape

**Table 3:** Inspection sequences of waiting-inspection targets in *k*th span segment.

Number	Line Number	Inspection Point	Coordinate in ECS	Class	Inspection Sequence
①	A-phase	A	A_G_	F1	LP1-LNA-GF1-ID1
②	Ground wire	B	B_G_	E3	LP1-LNG-GE3-ID2
③	C-phase	C	C_G_	E1	LP1-LNC-GE1-ID3
④	B-phase	D	D_G_	L1	LP1-LNB-GL1-ID4
⑤	A-phase	E	E_G_	F1	LP1-LNA-GF1-ID5

**Table 4:** Information of inspection targets on Lines 1–2.

Target	Relative Coordinate (m)			Preliminary Result	Inspection Sequence	PTZ Camera Pan/tilt		Image	Judgement
⑧	6.5	1.58	−0.11	GE2	LP1-LNA-GE2-ID1	−96.0	6.53	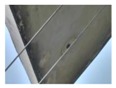	Y
⑦	5.62	0.74	−1.57	GL1	LP1-LNB-GL1-ID2	−93.6	5.20	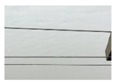	N
②	6.57	1.68	−5.22	GM1	LP1-LNA-GM1-ID3	−117.0	−18.10	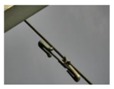	Y
④	5.37	1.75	−5.31	GM1	LP1-LNB-GM1-ID4	−122.7	−12.73	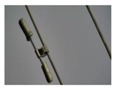	Y
⑤	6.65	1.95	−6.04	GE3	LP1-LNA-GE3-ID5	−130.0	−21.86	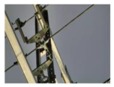	Y
⑥	5.64	1.93	−6.19	GE3	LP1-LNB-GE3-ID1	−135.2	−18.15	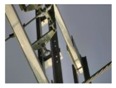	Y
①	6.74	1.84	−7.10	GM1	LP1-LNA-GM1-ID2	−147.9	−27.28	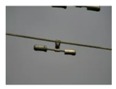	Y
③	0.76	1.89	−7.11	GM1	LP1-LNB-GM1-ID3	−152.2	−21.06	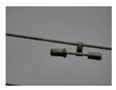	Y

**Table 5:** Inspection information of targets on three phase conductors.

Target	Relative Coordinate (m)			Preliminary Result	Inspection Sequence	PTZ Camera Pan/tilt		Image	Judgement
②	2.05	−12.4	−0.80	GM1	LP1-LNB-GM1-ID1	−26.1	49.3	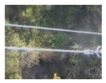	Y
①	0.55	−5.7	−0.85	GM1	LP1-LNA-GM1-ID2	−53.6	51.7	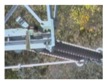	Y
③	1.05	−18.9	−0.88	GM1	LP1-LNC-GM1-ID3	−80.2	41.8	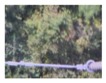	Y
